# Gut microbiome and gut-derived metabolites to identify the transition from metabolically healthy to unhealthy obesity: a mini review

**DOI:** 10.3389/fnut.2026.1989174

**Published:** 2026-10-14

**Authors:** Eugenia I. Manrique, Lawrence Sciglitano, Carmen P. Ortega-Santos

**Affiliations:** 1 School of Medicine, St. George's University, St George's, Grenada; 2 Department of Exercise and Nutrition Sciences, Milken Institute School of Public Health, The George Washington University, Washington, DC, United States

**Keywords:** cardiovascular disease, gut microbiome, metabolically healthy obesity, metabolome, obesity heterogeneity

## Abstract

Obesity is a metabolically heterogeneous condition with distinct metabolic phenotypes, ranging from metabolically healthy obesity (MHO) to metabolically unhealthy obesity (MUO). Although individuals with MHO initially lack cardiometabolic dysfunction, many transition to MUO over time, exponentially increasing their risk for type 2 diabetes and cardiovascular disease. In this mini-review, we synthesize recent evidence from multi-omic cohort studies evaluating gut microbiome composition and gut-derived metabolites as functional biomarkers of obesity heterogeneity and drivers of metabolic decline. While microbial diversity yields inconsistent signatures across cohorts, MHO is generally characterized by higher relative abundance of Ruminococcaceae, *Oscillospira, Akkermansia*, and *Bifidobacterium* and enrichment of other taxa predicted to be short-chain fatty acid-producers. Conversely, circulating gut-derived metabolomic signatures including altered secondary bile acid pools, elevated branched-chain amino acids, and trimethylamine N-oxide more reliably discriminate MUO from MHO than traditional measures, and correlate strongly with visceral adiposity, insulin resistance, and systemic inflammation. Furthermore, new systems biology approaches, such as integrating high-dimensional metagenomics and metabolomics via machine learning models, offer a potentially superior early indicators tool in precision medicine for identifying obesity phenotypes compared with traditional metrics (e.g., body mass index, fasting glucose). Overall, current evidence remains predominantly cross-sectional and based on secondary analyses of large cohort studies, underscoring the need for prospective longitudinal studies to validate these multi-omic biomarkers for precise phenotype identification and clinical risk assessment.

## 1 Introduction

Obesity is a heterogeneous, multi-factorial, and complex disease, with individuals exhibiting significant variation in metabolic health and consequently disease progression (1). Growing clinical and scientific evidence has emphasized the importance of defining this heterogeneity to improve cardiovascular disease (CVD) prevention and treatment (2). In particular, a major unmet clinical challenge is predicting when and which individuals will transition from metabolically healthy obesity (MHO) to metabolically unhealthy obesity (MUO), a shift associated with increased cardiometabolic conditions and CVD risks (3, 4). Emerging evidence suggests that the composition of the gut microbiome and gut-derived metabolites may serve as biomarkers of obesity phenotypes and early identifiers of those at higher risk of transitioning from MHO to MUO. In this mini-review, we synthesize current evidence on gut microbial taxa, functional pathways, and circulating and fecal metabolites and help identify individuals at increased risk of transitioning from MHO to MUO. This approach could enhance our understanding of obesity by shifting the focus from a *one-size-fits-all* entity to a range of biologically distinct phenotypes with unique health trajectories. We identified literature for this manuscript through a structured search in peer-review biomedical databases (e.g., PubMed, Google Scholar) between July 2024 and August 2026. The search strategy employed keywords including *gut microbiome, microbial metabolome, metabolome, obesity phenotypes, obesity heterogeneity*, and *cardiovascular disease*. We targeted peer-reviewed studies published within the last 5 years that used 16S rRNA sequencing, metagenomics, metabolomics, and multi-omics integration to classify metabolic obesity phenotypes. When recent data were limited for specific subtopics, we extended the search window to 10 years.

## 2 Metabolic heterogeneity in obesity: from MHO to MUO

A central framework for understanding obesity heterogeneity is classifying individuals by cardiometabolic health status (2). Although diagnostic criteria vary across studies, individuals are commonly categorized into four obesity phenotypes based on body mass index and the presence or absence of cardiometabolic risk factors (e.g., fasting glucose, blood pressure, blood lipids) according to NCEP-ATP III guidelines (1): metabolically healthy normal weight (MHNW), metabolically unhealthy normal weight (MUNW), metabolically healthy obesity (MHO), and metabolically unhealthy obesity (MUO). Given this mini-review's focus on obesity phenotypes and the transition from MHO to MUO, this review does not discuss MHNW and MUNW in more detail. Interested readers should consult recent reviews on these phenotypes (5).

MHO is generally characterized by the absence of major metabolic abnormalities despite excess adiposity, whereas MUO is associated with the presence of at least one or more of the following disturbed health conditions: insulin resistance, dyslipidemia, hypertension, and systemic inflammation (2, 6, 7). However, MHO is sometimes described as a transitional rather than stable phenotype along the continuum of cardiometabolic dysfunction (8). Longitudinal studies indicate that a substantial proportion of individuals with MHO eventually progress to MUO, although the timing of this transition is highly heterogeneous, ranging from only a few years in some individuals to more than a decade in others (2, 8, 9). This variability suggests that the biological processes underlying metabolic deterioration begin well before cardiometabolic abnormalities are clinically apparent. Although widely used, the MHO-to-MUO framework may oversimplify obesity's biological complexity. A recent preprint describes additional obesity phenotypes, identifying up to 11 distinct subtypes and underscoring the need for biomarkers that capture underlying biological and metabolic heterogeneity and better identify individuals at risk for adverse metabolic and cardiovascular outcomes (10). Precision nutrition studies show that individuals with obesity exhibit distinct tissue-specific metabolic phenotypes, such as predominant liver vs. muscle insulin resistance, representing biologically divergent pathways to metabolic deterioration (11). Overall, the marked heterogeneity in obesity metabolic transition trajectories emphasizes the need for biomarkers that can identify individuals with obesity at different stages of metabolic and cardiovascular deterioration before disease onset. Among the candidate biomarkers currently under investigation, gut microbiome composition and gut-derived metabolites have emerged as promising indicators of metabolic health status and disease progression (12).

## 3 Gut microbiome composition and obesity phenotypes

Over the past two decades, the gut microbiome has been proposed as a relevant contributor to the etiology of obesity and progression to CVD (13, 14). Beyond its proposed role in obesity pathogenesis, microbial composition may clarify obesity heterogeneity by distinguishing distinct phenotypic profiles and identifying biomarkers predictive of the transition from MHO to MUO. However, current evidence on microbiome diversity across obesity phenotypes remains inconsistent. Several studies have reported higher alpha diversity in individuals with MHO than in those with MUO (15, 16), while others have found no significant differences between these phenotypes (17–20). These cross-study discrepancies may partly stem from population-level heterogeneity, including geographic and demographic differences across cohorts, as well as variations in underlying microbial community structure. To explain these discrepancies, Lee et al. identified two microbiome-derived enterotypes within MHO, dominated by either *Akkermansia* or *Prevotella*. While individuals within the *Akkermansia* enterotype demonstrated significantly higher microbial alpha diversity, those within the *Prevotella* enterotype did not, suggesting that taxonomic composition may provide greater discriminatory value than diversity metrics alone (16). Such findings highlight the importance of considering community structure when evaluating microbiome-based signatures of metabolic health. Furthermore, this inconsistency may partly result from confounding between adiposity and metabolic status. In a cohort that separated the two, obesity measures explained most variation in microbiome diversity, with few features linked solely to metabolic health (21), suggesting that poor metabolic health may mainly exacerbate the diversity loss caused by obesity.

In contrast, differences in microbial taxonomic composition and inferred microbial functional activity appear to be more consistently associated with metabolic health status. Recent studies employing advanced analytical approaches, including microbial network analyses and multi-omics integration, have identified distinct microbial signatures associated with MHO and MUO. MHO is consistently characterized by enrichment of predicted to be short-chain fatty acid (SCFA)-producing taxa, including members of Ruminococcaceae, *Oscillospira, Akkermansia*, and *Bifidobacterium* (15, 18, 22). These microorganisms have been linked to improved gut barrier function, reduced inflammation, and favorable metabolic regulation, suggesting a potential role in preserving metabolic health despite excess adiposity (23–26). Conversely, studies of obesity phenotypes indicate that MUO is associated with enrichment of microbial taxa linked to inflammation, impaired glucose homeostasis, and dysregulated lipid metabolism (17, 19, 27). Notably, several taxa depleted in MUO, including predicted SCFA-producing organisms such as *Akkermansia, Oscillospira*, and members of the Ruminococcaceae family, overlap with taxa consistently reported to be reduced in CVD (28). This observation raises the possibility that loss of metabolically protective microorganisms may represent an early microbial feature linking the transition from MHO to MUO with increased CVD risk before the presence of traditional cardiometabolic deleterious biomarkers (e.g., increased fasting glucose, disrupted blood lipids).

While several studies have identified taxa associated with either MHO or MUO, focusing solely on individual microorganisms may overlook broader community alterations within the gut microbiome. The driver-passenger model, originally proposed to explain sequential bacterial involvement in colorectal cancer (29), has recently been adapted and extended to cardiometabolic disease by Wrigley et al. (18): highly pathogenic “driver” taxa are proposed to initiate disease processes and remodel the host gut ecosystem, creating conditions in which better-adapted “passenger” taxa outcompete other community members, including the drivers themselves, for resources. Notably, both Wrigley et al. (18) and Lacruz-Pleguezuelos et al. (19) reported evidence of altered microbial network architecture in metabolically unhealthy states, characterized by reduced connectivity and loss of key microbial interactions. Collectively, these findings support a conceptual framework in which progression from MHO to MUO is characterized by the gradual loss of metabolically protective taxa, enrichment of pro-inflammatory microorganisms, and disruption of microbial network architecture (Figure 1). As these functional shifts are not always captured by composition alone, gut-derived metabolite signatures may offer an additional, more direct functional readout of ecosystem function across the MHO-to-MUO transition.

**Figure 1:**
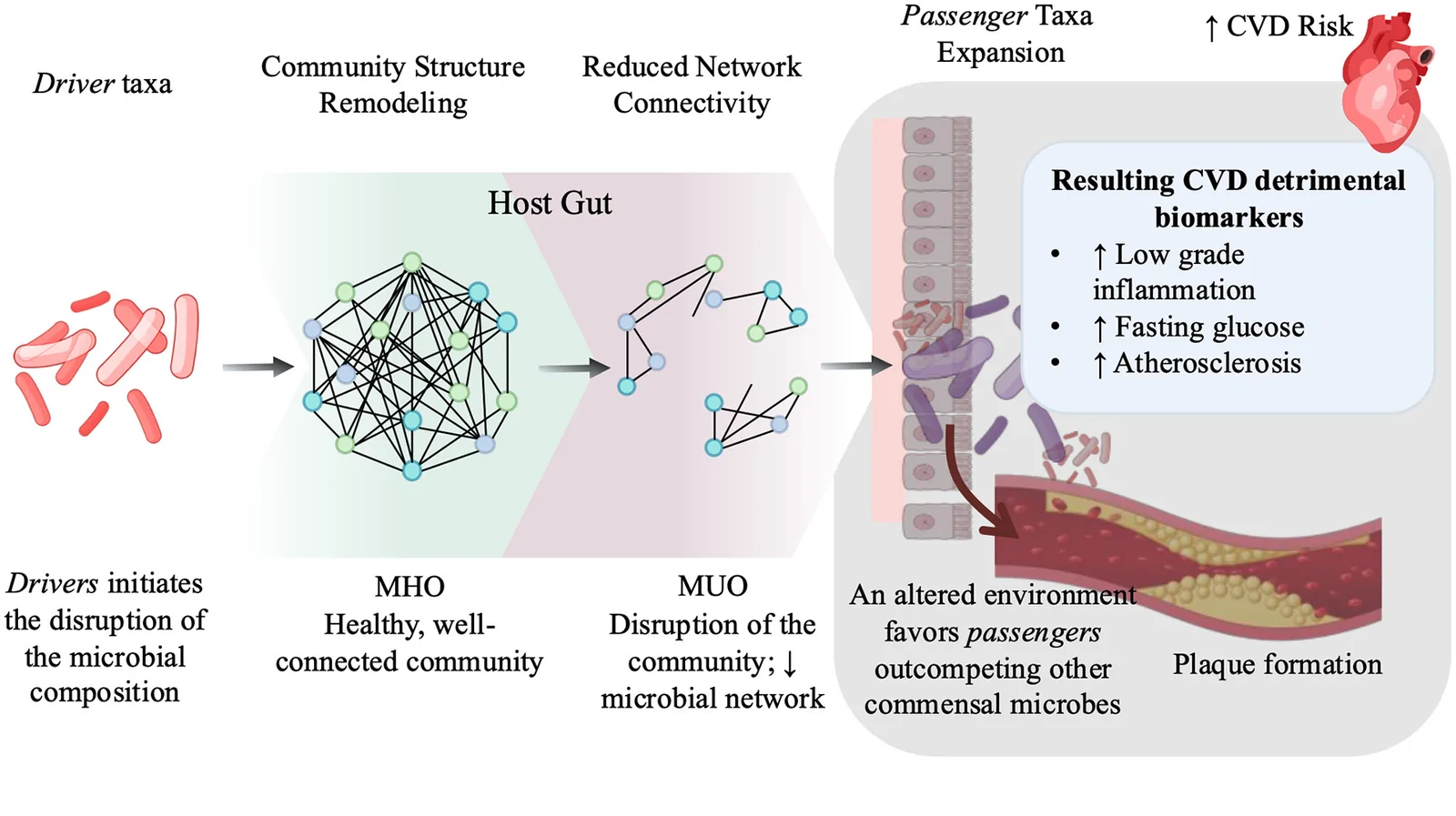
Conceptual framework of gut microbiome alterations during the transition from metabolically healthy obesity (MHO) to metabolically unhealthy obesity (MUO). The progression is characterized by ecosystem remodeling, potentially initiated by pathogenic “driver” taxa, leading to the gradual depletion of metabolically protective taxa and the subsequent enrichment of better-adapted, pro-inflammatory “passenger” microorganisms. Concurrently, this transition disrupts overall microbial network architecture, marked by reduced community connectivity and the loss of key inter-microbial interactions. CVD, cardiovascular disease. Adapted from the driver-passenger model originally proposed for colorectal cancer (29), as applied to cardiometabolic by Wrigley et al. (18).

## 4 Gut-derived metabolites as biomarkers of the transition from MHO to MUO

Numerous studies have summarized metabolomic signatures associated with obesity, cardiometabolic health, and vascular status (30–32). Reduced fecal SCFAs, including butyric acid, are consistently linked to obesity and unhealthy cardiometabolic phenotypes (27, 33–35). Circulating metabolomic profiles further distinguish MHO from MUO, with MUO characterized by higher levels of branched-chain amino acids (BCAAs) and less favorable fatty acid profiles, alongside lower plasma levels of gut-derived propionate (34). Additionally, elevated circulating cholic acid has been observed in metabolically unhealthy non-obese individuals (36), supporting a potential role for bile acid (BA) metabolism in cardiometabolic dysfunction that warrants further investigation in MHO and MUO populations.

Nonetheless, findings on gut-derived metabolites remain limited and conflicting across cohorts (35), likely driven by differences in study design, biological matrices, and analytical platforms. Consequently, the precise role of microbial metabolites in driving the MHO-to-MUO transition remains incompletely understood. Given this scarcity of mechanistic data, gut microbiota-derived metabolites (37), including trimethylamine N-oxide (TMAO), 3-phenylpropionate, and secondary BAs, represent critical candidate intermediates linking gut microbial composition to cardiometabolic disease and CVD risk.

TMAO, generated through microbial metabolism of dietary choline and carnitine, has been associated with atherosclerosis, CVD, and metabolic dysfunction (38, 39). Secondary BAs, produced through microbial transformation of primary bile acids, influence glucose, lipid, and energy metabolism through host signaling pathways (40). In addition, microbial phenolic metabolites such as 3-phenylpropionate have been associated with greater gut microbial diversity and favorable metabolic profiles, suggesting a potential role in cardiometabolic health (41). Collectively, these findings support the concept that gut microbiota-derived metabolites may contribute to the transition from MHO to MUO. However, despite growing evidence of their biological relevance to cardiometabolic health, researchers have rarely investigated these specific microbial signatures as predictive biomarkers for the loss of metabolic health in obesity.

Characterization of the gut metabolome has shown potential to serve as a predictive biomarker, a measure of treatment response, and an indicator of disease progression, in addition to its promising role in disease diagnosis (42, 43). Fecal metabolomics has emerged as a promising approach to directly assess the gut microbiome's functional output and its potential contribution to metabolic health (44). Furthermore, the circulating metabolome explains ~60% of the variation in gut microbiome composition (45–47), offering a more accessible window into microbial activity. Thus, integrating fecal and circulating metabolomes captures gut-derived microbial signals and provides a robust readout of host-microbe metabolic crosstalk across different obesity phenotypes.

Importantly, fecal and circulating metabolomes provide complementary information. Whereas fecal metabolites reflect microbial activity within the intestinal lumen, circulating metabolites represent the fraction of microbial and host-derived compounds that enter systemic circulation and may directly influence peripheral tissues and cardiometabolic health (33, 44). Integrating these two metabolomic compartments with gut microbiome data may therefore enable the identification of microbial metabolic pathways that are locally active in the gut and simultaneously relevant to host physiology. This approach could improve biomarker and mechanism detection in obesity heterogeneity, while helping distinguish metabolites that merely correlate with microbial composition from those that may mediate the transition between MHO and MUO.

## 5 Integration of microbiome and metabolomics: toward functional biomarkers of metabolic health

Recent multi-omic investigations have begun to shed light on the complex interactions among gut microbial composition, microbial metabolism, gut-derived metabolites, and host metabolic health, highlighting the value of integrated approaches for identifying mechanisms and biomarkers underlying obesity heterogeneity.

Watanabe et al. (48) demonstrated that a metabolomics-informed BMI model captures obesity-related metabolic heterogeneity more effectively than BMI alone, identifying a panel of metabolites associated with MHO and MUO signatures, including several metabolites of putative gut origin such as glutamate, mannose, N-acetylglycine, and 3β-hydroxy-5-cholestenoate. In a subsequent and larger multi-omic investigation, Chakaroun et al. (49) extended these observations by integrating metabolomics, metagenomics, and deep clinical phenotyping, demonstrating that a metabolome-informed BMI signature was strongly associated with visceral adiposity, insulin resistance, and reduced gut microbial diversity independent of measured BMI. Notably, most metabolites in this signature were predicted by gut microbial species, and mediation analyses identified numerous microbiome-to-phenotype pathways operating through circulating metabolites. Similarly, large-scale machine learning (ML) analyses in deeply phenotyped cohorts demonstrated that specific gut microbial species are strongly tied to baseline circulating metabolic markers, liver fat accumulation, and systemic inflammatory profiles (50). Together, these studies provide preliminary converging evidence that gut microbiota-derived and gut microbiota-modified metabolites may help explain obesity heterogeneity and distinguish metabolically healthy from unhealthy individuals beyond conventional anthropometric measures. Moreover, they highlight the potential of integrated microbiome-metabolome profiling for identifying individuals at risk of transitioning from MHO to MUO before overt clinical manifestations emerge.

## 6 Conclusion and future directions

Taken together, the evidence reviewed here indicates that gut microbiome composition and gut-derived metabolites are consistently associated with metabolic health status in obesity, distinguishing MHO from MUO beyond what BMI or traditional cardiometabolic risk factors alone capture. Depletion of SCFA-producing taxa, disruption of microbial network architecture, and shifts in fecal and circulating metabolites (including SCFAs and other gut-derived compounds such as TMAO and secondary BAs) each appear to track metabolic deterioration, yet they remain underutilized as tools to identify the specific transition from MHO to MUO. However, the current evidence base remains largely cross-sectional, compositional rather than functional, and rarely integrates microbiome and metabolome data within the same cohort. As a result, it is not yet possible to determine whether these microbial signatures drive, result from, or simply correlate with the transition from MHO to MUO, or whether they precede clinically apparent cardiometabolic and cardiovascular dysfunction.

Addressing these gaps will require a shift in study design toward more precise obesity phenotyping, direct microbiome functional measurement, longitudinal, multi-omic, and experimental approaches using a systems biology approach that can establish temporality and causality rather than association. Table 1 summarizes the suggested directions we consider most likely to advance the field, spanning more precise classification of obesity phenotypes (including longitudinal MHO-to-MUO transitions), direct quantification of fecal and circulating metabolites, integration of microbiome and metabolome data to test whether specific taxa or pathways explain metabolite differences, and downstream cardiovascular phenotyping beyond traditional risk factors. Critically, we highlight the need for multi-omic study designs that link the full pathway from metagenomics to microbial pathways, fecal metabolites, circulating metabolites, and host phenotype, coupled with experimental validation of candidate metabolites to move the field from association toward mechanism. Pursuing these directions could clarify whether gut microbiome and metabolome profiling can serve as clinically actionable biomarkers to identify individuals at risk of transitioning from MHO to MUO before overt CVD develops.

**Table 1:** Future directions.

#	Future direction	Rationale
1	Refined obesity phenotyping frameworks	Move away from static MHO vs. MUO toward continuous risk scores and multi-dimensional phenotypes (e.g., fat distribution, ectopic lipid accumulation) that capture dynamic transitions across the obesity heterogeneity spectrum.
2	Paired fecal and blood metabolomic profiling	Concurrently quantify fecal and circulating metabolites to distinguish localized fecal output from systemically bioavailable, host-interacting molecules.
3	Implement prospective longitudinal designs	Track individuals over time to establish temporality and determine whether specific metabolomic shifts precede (and predict) metabolic breakdown, overcoming cross-sectional limitations.
4	Include advance cardiovascular profiling	Include direct measures of cardiovascular health such as endothelial function, arterial stiffness, subclinical atherosclerosis, or incident cardiovascular events, rather than relying solely on traditional risk factors (e.g., lipids, blood pressure).
5	Integrate multi-omic layers mechanistically	Use a systems biology approach to integrate metagenomics → functional pathway analysis → fecal metabolomics → circulating metabolomics → host phenotype to map an end-to-end causal pipeline instead of evaluating omic layers in parallel.
6	Apply AI/ML to guide experimental validation	Leverage machine learning (ML) algorithms and Artificial Intelligence (AI)-driven computational tools (e.g., digital twin technology) to identify gut-derived signatures candidates to identify gut-derived signature candidates to classify distinct obesity phenotypes and predict elevated CVD risk.
